# A Comparative Study of Commercial 3D Printers for Food Fabrication Based on Printing Ink Gels

**DOI:** 10.3390/gels12090777

**Published:** 2026-08-31

**Authors:** Ying Xu, Wenbo Xu, Jingfu Pan, Xiaochen Chen, Lin Lin, Yulin Zhu, Rentai Song

**Affiliations:** 1 College of Life Science, Yantai University, Yantai 264005, China; 2 School of Food and Biological Engineering, Jiangsu University, Zhenjiang 212013, China

**Keywords:** 3D food printing, extrusion, food ink gels, printer, nutrients

## Abstract

The increasing awareness of dietary health among residents has pushed the development of the food industry towards the field of personalized, nutrition-balanced and specific foods to cater to the demands of special populations, including children, elders, obese people and vegetarians. In recent years, 3D food printing technology has gained increasing attention due to its great potential in the preparation of personalized and nutrition-balanced foods. In this study, four types of 3D food printing technology, including extrusion-based printing technology, inkjet printing technology, binder jet printing technology and selective laser sintering printing technology, are illustrated in detail to compare their mechanisms, procedures, merits and applications. Among these 3D printing technologies, extrusion-based printing technology is dominant over other types of 3D food printing technologies. In addition, various commercial 3D printer devices based on different printing ink gels are presented in detail, and their application in different food preparations, such as chocolate, biscuits, candy, and coffee, is demonstrated, indicating the status and future of 3D food printing technology. In conclusion, 3D food printing is a promising direction for future trends of food development, allowing for the manufacture of personalized and nutrition-balanced food, diversification of food production and co-participation in food preparation.

## 1. Introduction

The sudden outbreak of COVID-19, along with global climate change and population aging, has profoundly reshaped the food industry landscape in recent years. Pandemic-related disruptions have manifested as supply chain bottlenecks, labor shortages, shifts in consumer purchasing behavior, and increased food loss, all of which have collectively influenced consumption patterns, purchasing power, and dietary habits. These challenges have prompted the food sector to seek innovative technological solutions that can enhance resource efficiency, adapt to changing consumer demands, and improve the resilience of food production systems. In this context, 3D food printing has gained increasing attention and emerged as a promising area of research and development. This technology enables the fabrication of food products using a variety of compositional gel materials, offering notable potential in the preparation of easy-to-chew foods, personalized nutrition, and customized multi-nutrient formulations. Beyond these direct product-oriented advantages, 3D food printing also possesses inherent characteristics that may contribute to the diversification of food production, participatory food preparation, and the socializing of manufacturing processes, potentially supporting economic growth and employment. However, it is important to recognize that the technology is still in its early stages, with significant limitations in printing speed, material diversity, and final food quality that currently constrain its large-scale deployment. Nevertheless, as a decentralized and flexible manufacturing approach, 3D food printing represents a forward-looking direction that aligns with the post-pandemic emphasis on supply chain adaptability and localized production, warranting continued research and development efforts [1].

Relevant research on the application of 3D printing technology in the food field is quite ample, mainly focusing on active food packaging [2,3,4,5]. There are studies that have reported that 3D printing is a viable technology for manufacturing intelligent components that can be integrated into conventional food packaging to create intelligent food packaging (IFP) [6], such as hydrogel–oleogel–bigel-embedded purple sweet potato anthocyanin (PSPA), extruded on food film through 3D printing to form a composite film as a color-resistant and leach-resistant volatile amine sensor [7]. Food ingredients such as proteins, fats, and sugars, which serve as printing ink gels, play a crucial role in the final quality of products. Before the introduction of food inks, similar bio-inks were used in 3D bioprinting and showed extraordinary talents. As early as 2010, there were systems using inkjet and gel technology with inkjet droplets as bio-compatibilizers to embed cells and use them in direct 3D cell printing systems [8]. Nowadays, methylcellulose [9], chitosan [10], alginate [11], etc., are often used in bio-inks for 3D bioprinting by mixing with other water–oil components or colloids, such as gelatin [12], xanthan gum, gum arabic, etc., to form hydrogels. In addition, hydrogels based on nucleotide lipids [13] or proteins/peptides [14] are new bio-ink materials in biofabrication. Some bio-inks, such as methylcellulose, have also often been used in the food field to maintain the freshness of food due to their stability and film-forming properties [15,16,17,18]. As for food inks, five categories are reported, namely confectionery, dairy, hydrogels, plants, and meat [19]. Of these five categories of food inks, protein compositions are dominating; thus, it is widely considered that protein may be the ideal 3D printing ink for foods [20]. Nowadays, 3D food printing using proteins as printing ink gels has achieved some progress. For example, plant protein is mostly used to make plant protein-based meat products via 3D printing to achieve the replacement of animal meat products [21]. As for animal proteins, several special 3D food products printed with animal proteins have been optimized and commercialized for special populations (dysphagia patients, laryngeal carcinoma patients, etc.) with special dietary requirements and have proven to be viable options with good success [22].

Apart from proteins, other food nutrients such as polysaccharide, lipids and microelements are also incorporated into food ink gels to meet the personalized requirements of consumers, which provides a platform for research on mixed inks containing a variety of nutrients [23]. To date, studies on starch polysaccharide-based mixed inks account for the vast majority of the literature [24,25,26]. However, one aspect that cannot be overlooked for the future development of mixed food ink gels in 3D printing is the global acceptability of this emerging and innovative continuous food processing technology [27]. Unlike previous reviews that focus solely on ink formulation or a single printing principle, this review systematically compares commercially available 3D food printers across four major technological categories, with an emphasis on the interplay between printer hardware and food gel rheology. We provide a practical guide for researchers and industry practitioners to select appropriate equipment based on product requirements, and we critically assess the gap between current commercial capabilities and the idealized vision of personalized nutrition. By integrating equipment specifications, material properties, and post-processing considerations, this review offers a holistic framework that is directly actionable for food product development.

## 2. Types of 3D Food Printing Technologies

### 2.1. Extrusion-Based Printing Technology

**Figure 1:**
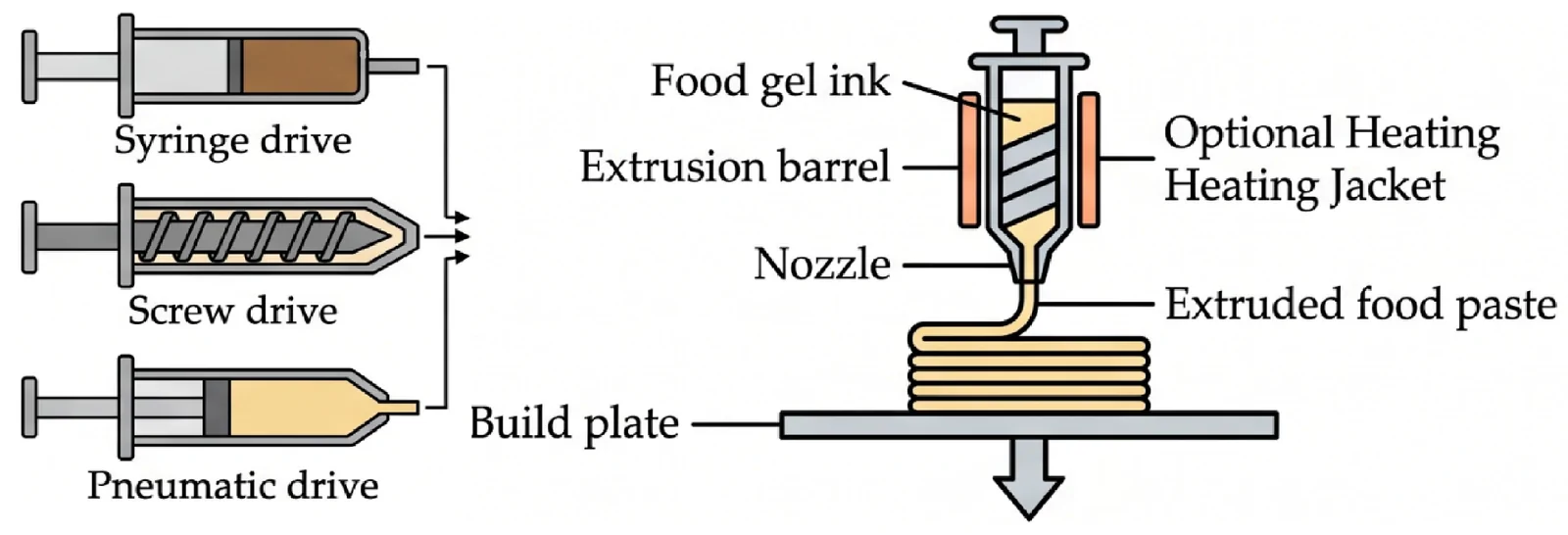
Extrusion-based 3D food printing [28].

Extrusion-based printing technology is currently widely employed in 3D food printers. This burgeoning technology uses ductile, slurry, or pasty materials, such as liquids or pastes, that are loaded into a syringe and forced out through a moving nozzle fixed to the printer bed; the extruded material is then deposited and stacked while cooling. Soft ingredients commonly used in extrusion-based printing include cheese, meat paste, dough, mashed potatoes and chocolate. Three extrusion mechanisms are applied in this technology: screw-based, syringe-based and air-pressure-based extrusion (Figure 1).

In syringe-based extrusion, a syringe tube holds the printable material, and a plunger driven by a stepper motor is put inside this syringe tube. The stepper motor enables a rectilinear movement of the plunger, which pushes the material toward the nozzle tip and extrudes it. Semi-solid materials such as chocolate, ice cream and dough are well suited to this printer type [29,30,31]. Similar to the plunger concept, the use of a piston in syringe-based extrusion has been successfully optimized with respect to head travel speed and piston pressure for food materials of varying viscosities [32].

In screw-based extrusion, the print components are mixed separately before being fed into a filter element that rotates via a motor. The materials are then driven through the tube by a screw thread and extruded onto the print bed through the nozzle tip. This technology is commonly used for the 3D printing of chocolate [33]. However, screw-based 3D printers have been reported to be unsuitable for extruding high-viscosity ink gels [28]. Gel-like substances with high mechanical strength and highly viscous materials are not appropriate for screw-based printing, as weak interlayer adhesion can cause compression, deformation and low resolution during the printing process [34].

In air-pressure-based extrusion, air pressure is generated by a dispenser, and the printable material is placed in a barrel from which compressed air forcefully pushes it to the nozzle tip and onto the print bed. This technique first appeared in bio-3D printing for precision ink deposition and has now been adopted for dispensers in the food sector [35]. Because food ink gels contain a variety of nutrients, leading to complex physical properties such as variable viscosity, dispensers will remain in use in the food field for the foreseeable future.

In addition, other extrusion systems are occasionally used, such as peristaltic pump-based extrusion [36] and gear-based extrusion [37], which can effectively address extrusion flow issues. However, condensable ink gels like chocolate are better suited to hot extrusion [38]; their extrusion system resembles fused deposition modeling (FDM) used in the machinery industry, with a separate heating sleeve around the ink barrel. Unfortunately, this system is costly and unsuitable for multi-nozzle setups. Extrusion-based 3D printing offers many advantages, including a simple principle, low cost, and compatibility with a wide range of inks. Nevertheless, some extrusion systems lack sufficient extrusion capacity, leading to reduced product quality. To improve product quality, novel strategies have been explored, such as reducing nozzle diameter to the micron scale [39] and combining machine vision with feed-forward motion control to achieve consistent extrusion at each point [40]. Research trends in print quality characterization are becoming increasingly evident, and enhancing the quality and efficiency of printed products remains an ongoing challenge. A previous study demonstrated that applying certain physical fields to pretreat printing materials can effectively improve printability and accuracy during the printing process [41].

### 2.2. Inkjet Printing Technology

**Figure 2:**
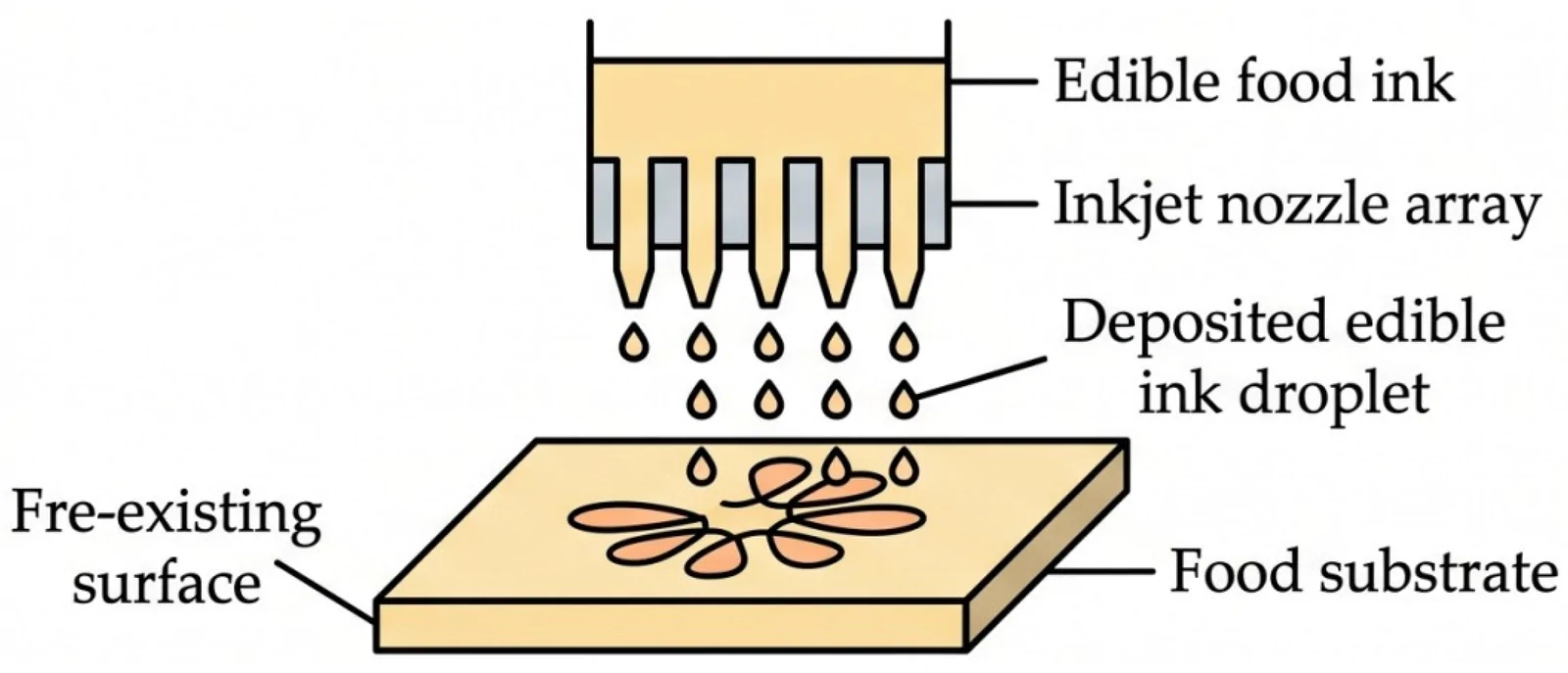
Inkjet-based 3D food printing [44].

Inkjet printing ejects a stream of droplets from a thermal or piezoelectric printhead onto specific areas for surface filling or image decoration on food surfaces. The thermal head heats the gels in the nozzle, causing them to expand and then be extruded through an electrothermal element [42]. In contrast, the piezoelectric head contains a piezoceramic component that generates electricity through its distortion (Figure 2). Under the control of a preset program, droplets are generated and deposited onto the substrate, gradually accumulating to form a 3D pattern, which contributes to the creation of images on food surfaces [43].

Based on droplet generation processes, inkjet printing can be classified into two types: continuous inkjet printing (CIJ) and drop-on-demand printing (DDP). In CIJ, the ink gels are forced out of a nozzle as a consecutive liquid stream, which then breaks into droplets due to interfacial tension. A piezoelectric transducer is commonly incorporated in the nozzle assembly. By adjusting the transducer’s output frequency, the liquid flow through the nozzle can be optimized, thereby improving the dispersion of the liquid material [45]. Since the resulting droplets are usually electrically charged, a previous study reported a method to improve chocolate printing accuracy by varying the applied voltage to identify three discharge states [46]. In DDP, however, the printing equipment typically has multiple nozzles, enabling color printing decoration of foods. Because the printing material is ejected only from selected nozzles as needed, no continuous liquid flow is formed.

Interestingly, one study revealed that 3D inkjet printing can produce foods with bite-to-bite contrast, which could be beneficial for designing healthier food products [47]. Various methods have been developed to characterize foods and enhance their taste in the context of inkjet-based food 3D printing. Piezoelectric inkjet, a conventional inkjet printing technology, is limited by the viscosity of the ink gels. This limitation has been overcome by methods such as pneumatic jetting for edible formulations, as demonstrated by Vadodaria and Mills [48]. In other fields, inkjet printing technology has been used to manufacture flexible sensors, including gas, biopharmaceutical, temperature and humidity sensors. Rapid detection of food quality and safety using flexible sensor technology will play a significant role in households, packaging centers, and manufacturing facilities [21]. Moreover, integrating flexible sensors with food packaging expands the application scope of inkjet printing and offers new avenues for food quality and safety researchers.

### 2.3. Binder Jetting Printing Technology

**Figure 3:**
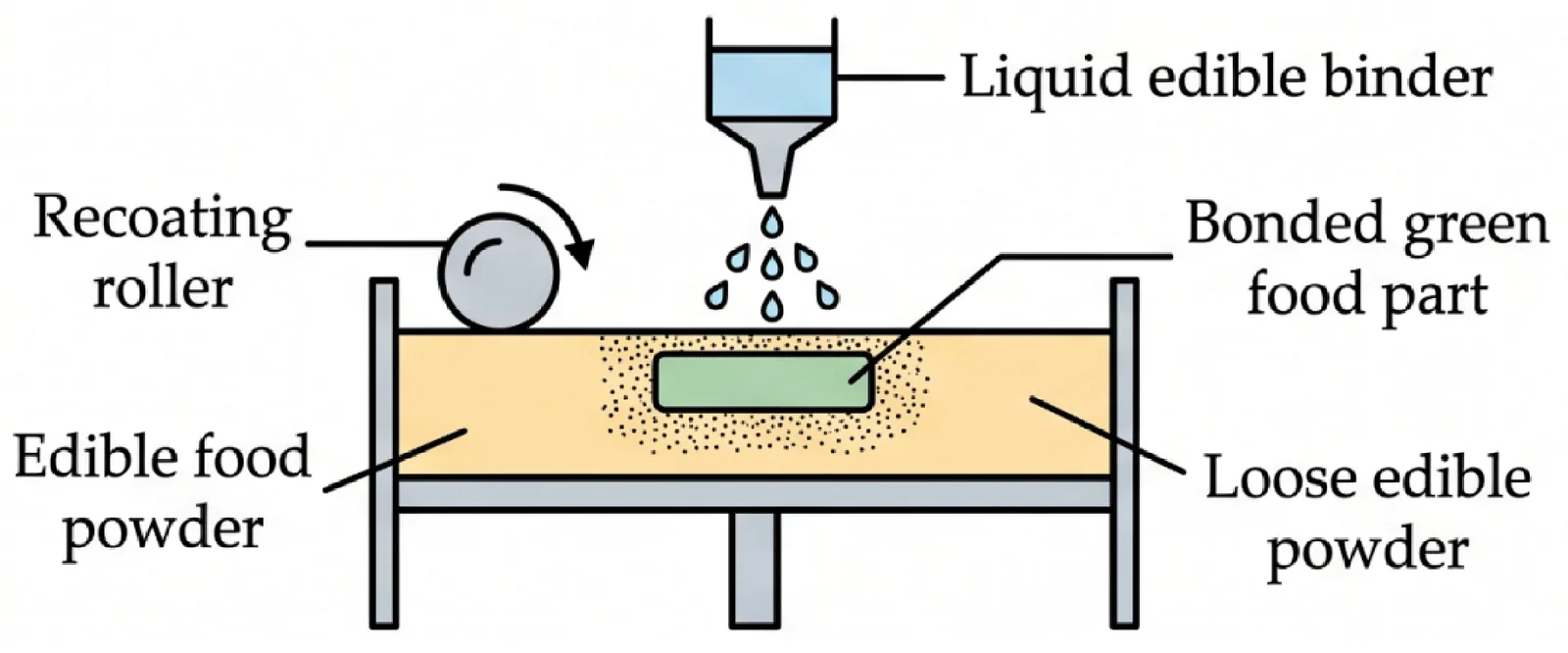
Binder jetting 3D food printing [45].

Binder jetting is a powder-bed-based additive manufacturing technique in which a liquid binding agent is selectively deposited onto a powder layer to bond particles into a solid structure, layer by layer. Its principle is analogous to that of powder-bed fusion processes such as selective laser sintering (SLS) but with a key difference: instead of using a laser as the energy source, binder jetting uses an inkjet printhead to deposit a liquid binder that chemically or physically binds the powder particles (Figure 3). The printing cycle begins with the printhead spraying binder along predetermined paths; a rotary roller then coats the next powder layer, and the build platform descends by one layer of thickness. This sequence repeats until the desired 3D object is formed [49,50]. Agglomeration is a well-known phenomenon in many commercially available products across different length scales, from rehydratable powdered beverages or soups to grain-based clusters [51]. Thus, binder jetting is an ideal choice for preparing edible ingredients.

Adhesives are essential for the bonding process. As natural adhesives, colloids are often added to food inks for 3D-printed food preparation. In a study by Holland et al. [52], xanthan gum and semi-crystalline cellulose powder were used as food inks in a binder jet printhead, demonstrating great potential for creating functional ingredients. In a subsequent study, the same authors added konjac glucomannan to the ink and verified the synergistic effect of cellulose, xanthan gum and glucomannan to the printed structure, providing a method for the structural reproduction of natural foods [53].

Recently, a study successfully applied binder jetting to print food products from a powder mixture of Calcium Caseinate (CaCas), starch, and medium-chain triglyceride (MCT) powder. This work enables researchers to create protein-rich foods with customized shapes, textures, colors, and novel tastes, thereby expanding the range of the food inks available for binder jetting printing [54]. In the pharmaceutical field, Wang et al. [55] demonstrated a useful case study for optimizing piezoelectric parameters of the printhead in the binder jet 3D printing of drugs, which can serve as a reference for parameter optimization in food binder jet printing.

### 2.4. Selective Laser Sintering Printing Technology

**Figure 4:**
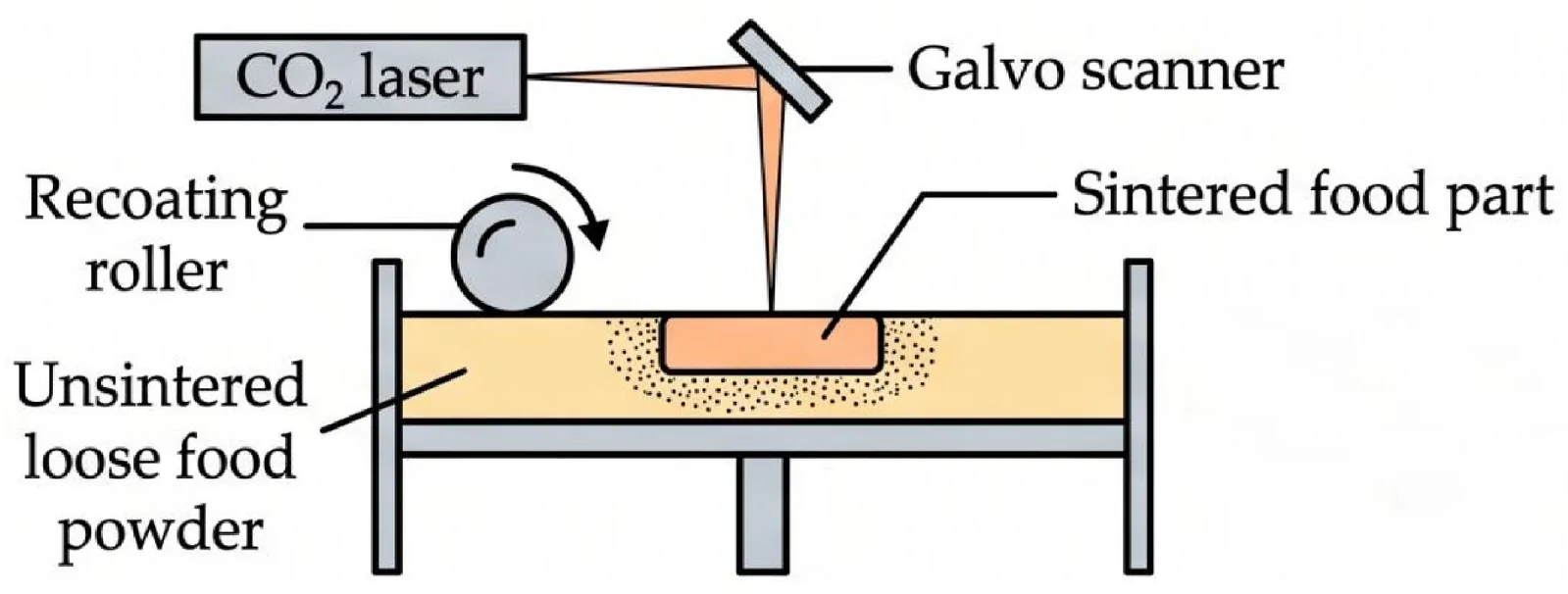
Selective laser sintering 3D food printing [57].

Selective laser sintering (SLS) typically uses infrared lasers as the energy source, and the raw materials are mostly metal or non-metallic powders. During processing, the powder is first preheated to a temperature slightly below its melting point and then leveled by a roller (Figure 4). The laser beam then selectively sinters the powder according to the path data for each layer, which is controlled by a computer program. After a layer is completed, the next layer is sintered, and excess powder is removed at the end of the process to form the final part. For food materials, because most food powders have lower melting points than metals, preheating is not required, allowing the use of various sintering sources such as selective laser sintering (SLS) and selective hot air sintering and melting (SHASAM) [56]. The advantage of selective sintering is that it is more convenient and faster, with no need for subsequent curing [45]. Moreover, selective sintering facilitates the production of nutrient-variable foods, thereby contributing to the development of functional foods.

Both SLS and SHASAM, which use laser or hot air to fuse powder materials in a layer-by-layer fashion, offer the great advantage of high efficiency for mass production [58]. However, these technologies are currently underutilized and have only been investigated for sugar or sugar-rich products [59]. In contrast, a pharmaceutical study by Thakkar et al. [60] demonstrated the first integration of a new continuous granulation technology with powder-bed-fusion-based selective laser sintering (SLS) process to improve the dissolution rate and physical characteristics of a poorly water-soluble drug, providing a method for manufacturing functional particles with enhanced properties.

Recently, Jonkers et al. [61] published several studies on selective laser sintered foods. In their work, they established a constitutive model describing the anisotropic elasto-viscoplastic damage behavior of the material. They then experimentally investigated the influence of process parameters on the microstructure and anisotropic mechanical properties of starch-based food produced by selective laser sintering, contributing to the understanding of their mechanical behavior [62]. To effectively reduce the warpage caused by the product size, they adopted the unit cell method [63]. Interestingly, another study applied selective laser sintering to fabricate magnetic sugar-based composites [64]. These composites can act as millimeter-scale robots, performing corkscrew movements in solutions with viscosity similar to biological fluids and can be rapidly dissolved in water while being magnetically controlled. In summary, selective laser sintering technology involves many controllable factors, which also points toward research directions for improving intelligence and product quality.

The rheological properties of food inks are decisive for each printing technology [56,58]. For extrusion-based printing, the ink must exhibit shear-thinning behavior (viscosity decreases under high shear during extrusion) and rapid structural recovery (high recovery rate of G′ after deposition) to maintain filament shape [19,20]. High yield stress is necessary to prevent slumping [37,38], and the loss factor (tan δ = G″/G′) should be <0.4 for good shape retention [26,39]. For inkjet printing, low viscosity (typically 2–20 mPa·s) and low surface tension are required to form stable droplets [46,48], and the ink must resist clogging [43,44]; G′ and G″ are less relevant because no solid structure is formed [47]. For binder jetting, the powder bed must exhibit good flowability, and the binder liquid must penetrate quickly [52,53]; binder viscosity is critical (similar to inkjet) [50,54]. For selective laser sintering, the powder must have a narrow melting range and good thermal stability [61,62]; the rheology of the molten phase affects coalescence and final porosity [60,63].

## 3. Printers for Various Kinds of Food Products

The commercial printers included in this review were selected based on the following criteria: (1) they are explicitly marketed for food printing; (2) their technical specifications are publicly available from the manufacturer or authorized distributors; and (3) they have been cited in the peer-reviewed literature or featured in industry reports. The commercial availability of each model was verified as of January 2026 (the cutoff date for our search). We acknowledge that the market for food 3D printers evolves rapidly; therefore, some models may have been updated or discontinued after this date.

### 3.1. Chocolates

As mentioned above, 3D printing of chocolate is feasible and can be performed using multiple types of 3D printing devices. However, only two types of 3D printers are commercially available for this purpose: extrusion-based printers and FDM-based printers [65]. The specifications and print quality of food 3D printers vary across manufacturers, as shown in Table 1.

All chocolate 3D printers support the G code file format. Most are simple cantilever structures that are easy to operate and do not require high technical expertise. The remaining chocolate printers employ a box structure, which protects the chocolate from external environmental disturbances during printing. In terms of chocolate appearance, simple 2D image accumulation no longer attracts customers; rather, products produced by 3D stacking of variable cross-sections are more appealing to consumers. Notably, the chocolate 3D printer from 3Desserts Graphiques features a robotic arm and a dual extruder, which can effectively improve production efficiency. In contrast, the dual extruder on the Felix Food 3D Printer has far fewer degrees of freedom than the 3Desserts Graphiques model. Interestingly, Park et al. [66] fabricated an extrusion printer device consisting of dual syringes, heaters, mixers, motors and linear guides, and successfully printed colored chocolate, providing a good precedent for color printing.

The quality of a 3D printer is evaluated based on product quality and production efficiency. In one study, the authors used Choc Creator 2.0 Plus, a commercial 3D chocolate printer from Choc Edge Ltd., to improve the quality and molding efficiency of 3D-printed chocolates by optimizing the printing thickness, velocity and overhang angle [67]. However, the quality of the 3D-printed chocolate products largely depends on the movement speed, extrusion rate and cooling rate of the printer [68]. The most important parameter affecting the quality of 3D-printed chocolates is the ambient printing temperature, as it ensures adequate cooling and coagulation of cocoa butter, which is necessary for print stability [69]. Other methods, such as electrostatic inkjet, are also applied to improve the quality of 3D-printed chocolates and enhance the accuracy of two-dimensional images [46,70]. Therefore, future research on commercial chocolate 3D printers should focus on optimizing product quality and improving production efficiency.

### 3.2. Cookies and Cakes

Two types of 3D printers—extrusion-based and inkjet-based—are commonly used for the preparation of cookies and cakes. Temperature is a key factor influencing the forming and expansion of cookie dough, cake and pizza [71]; accordingly, the production process for cookies and cakes in commercial 3D printers typically involves two steps: printing the gel materials inside the machine and post-processing outside the machine. However, some printers, such as those from Natural Machine, allow both steps to be completed in one machine. The food 3D printers for cookie and cake production from other manufacturers are listed in Table 2.

Owing to the characteristics of matrix materials, extrusion-based printing is the best choice for the 3D printing of cookies, cake and pizza. Except for printers from Natural Machine, which have heating chambers for post-processing, all other models are semi-open. In particular, XYZPrinting offers printers with touchscreens that can print single-material and three-material formulations. Most of the remaining printers are based on polylactic acid (PLA) filament printing. Interestingly, Micromake has pioneered parallel-arm 3D printing applications in the food sector, adapted from Delta technology.

Printing and post-processing abilities are two important parameters for evaluating a printer, and they are closely associated with the storage modulus G′ and loss modulus G″ of the material. One study fitted G′ and G″ into a model and found, through in-depth characterization, that a higher flow coefficient and lower-frequency sensitivity of the sample favored the printing and reprocessing capacity of cookie dough [72]. In terms of printing parameters, the main factors affecting cookie quality are baking temperature and time. Other parameters such as nozzle diameter significantly affect the texture properties of cookies, while the filling rate can control the shape (swelling and deformation) of 3D-printed cakes [73]; cookie geometry, infill density, and structural deformation are also correlated [30]. Furthermore, studies have shown that incorporating hydrocolloids into cookie dough can maintain its dimensional stability during baking [74], and increasing the proportion of xanthan gum prevents the reduction in mechanical strength of cookie dough during temperature changes, thereby avoiding deformation. The incorporation of hydrocolloids in 3D-printed cookies has also opened up new research directions, such as encapsulating aromas in microcapsules and releasing them via microwaves [29], as well as embedding soluble soybean polysaccharide with hydrocolloids to create easy-to-swallow foods [75].

### 3.3. Coffee

It is important to distinguish between true 3D food printing, which builds vertical structures layer by layer, and 2D surface decoration (e.g., latte art printing), which deposits edible inks only on the top surface of a beverage or solid food. In the coffee sector, most commercial “3D printers” are in fact 2D inkjet devices that produce images on coffee foam; they do not create three-dimensional edible objects. This distinction is crucial for terminological clarity; although they share the inkjet mechanism, true 3D inkjet printing requires sequential layering and often a support material. In this review, we include coffee printers due to their market prevalence, but we emphasize that they operate on 2D principles and should not be equated with extrusion-based or sintering-based true 3D fabrication.

Extrusion-based printing and inkjet printing are two common types for coffee 3D printing. Compared with the 3D printing of other foods, coffee printing technology is relatively mature and has an early start. However, it is limited to printing 2D images on the coffee surface using edible materials, with the aim of influencing visual perception, without considering material molding or accumulation [76]. Therefore, the principle of coffee 3D printing is relatively simple, and more commercial printers are available on the market, as shown in Table 3.

Since only 2D images are printed onto the coffee foam, resolution is particularly important and determines image quality. Among the models listed, the coffee 3D printer supplied by the MYJ can print color photos fully automatically, offering three selectable resolutions. Germany Colorato, on the other hand, offers two printers that print two portions of the coffee. Interestingly, to outperform its competitors, China ColorSun has adopted a unique shape design and added the capability to capture and print images instantly. Rainbow, meanwhile, has directly improved the printing productivity of their printer, not only in terms of printing speed (10 s of coffee) but also enabling color printing on four cups of coffee simultaneously. Of course, there are many other coffee 3D printers on the market, which are not exhaustively listed here.

Coffee printers have been widely adopted in office buildings, restaurants, airports and other settings, while the research and development of coffee 3D printers in the lab seem to have entered a quieter phase. Consequently, some studies have focused on the recycling and valorization of waste coffee ground (WCG), including oil extraction of active substances [77], incorporation of decolorized WCG (DWCGs) into a polylactic acid (PLA) matrix to meet the mechanical strength requirements of 3D-printed products [78], modification of DWCGs with polypyrrole (PPy) for use with PLA in 3D-printed solar evaporators with high photothermal conversion efficiency [79], and extraction of biochar from WCG via controlled pyrolysis for use as a reinforcement agent in epoxy resin [80]. In summary, coffee 3D printing technology has been applied in many areas and become increasingly mature.

### 3.4. Candies and Sugars

For candy, extrusion-based printing technology mainly applies to 3D preparation [81,82,83,84]. It is worth noting that chocolate also falls into the candy category, so most chocolate 3D printers are also capable of printing candies (including condensation candy). Table 4 lists the different 3D candy printers available on the market.

First, FoodBot developed the FoodBot D2 with a dual extrusion head based on the FoodBot S2, which greatly improved its production efficiency. The ChefJet Pro, on the other hand, has the advantage of being able to print colored candies to attract customers, although it has unfortunately been discontinued. Notably, the CandyFab 6000 is the only 3D candy printer that uses SHASAM principles, while the Magic Candy Factory is the world’s first 3D gummy printer.

Printing parameters, such as nozzle diameter, nozzle–substrate distance, extrusion speed and printing speed, play a decisive role in the quality and shape of 3D printing products [81,82]. In addition, structural design also affects the texture characteristics of the product [84]. To produce better products, commercial 3D candy printers are continually being optimized. Interestingly, a study by Le et al. [85] on the effect of polyols on the printing performance of food ink gels may pave the way for future food ink preparation for 3D printing. In the medicine field, the successful production of gummies from gelatin and hydroxypropyl methylcellulose (HPMC)-based hydrogels demonstrates that 3D printing is an efficient method for generating gummy drug formulations in various shapes and colors [83]. Another study applied 3D printing to prepare stereolithography (SLA)-based capsaicin candies for the treatment of canker sores [86]. Overall, commercial 3D candy printers have matured, and current laboratory research trends are focused on the effect of food ink composition on 3D-printed products.

### 3.5. Meat

For meat 3D printing, since meat and its by-products are inherently fibrous and non-printable [36], it is necessary to modify their viscoelastic and mechanical properties by adding flow enhancers to obtain an extrudable paste-like material [87]. Liu et al. [36] developed a printer with peristaltic pumps to squeeze out slurries of fibrous meat and gelatin; this printer can use fibrous meat as a printing material and mitigate the solid–liquid separation issues associated with fibrous meat materials.

However, it is essential to ensure the hygiene quality (both pathogenic and physicochemical) of these products [88]; therefore, printers require continuous temperature monitoring throughout the feeding system, hopper, nozzle and platform, as well as an environment free from other chemicals. For temperature control, Portanguen et al. [89] described and discussed the development of a printhead (with flow rate and temperature control) and modifications to the print plate (temperature control). In another study, Ko et al. [90] successfully used a coaxial 3D printer to print meat analogues with excellent texture and elastic strength compared to that of beef, providing a new approach to address the hardness of printed meat. However, the production of 3D-printed meat products currently involves particle size reduction and dilution of meaty and savory flavors, which are generally associated with a reduction in meat product quality [22].

Currently, increasing research attention is being directed toward 3D-printed cultured meat [91,92,93,94], owing to its advantages such as energy savings, environmental protection, and high production flexibility. Cultured meat is defined as meat produced sustainably under laboratory conditions without animal sacrifice or excessive use of antibiotics [92]. In recent years, growing interest has been devoted to developing alternative technologies to conventional meat production, and cultured meat has come into the awareness of consumers, with commercialization potentially attainable in the near future. Nevertheless, the generation of textured, unprocessed meat remains a major challenge. Thus, major emphasis should be placed on developing sustainable in vitro meat culturing on a commercial scale [95]. Notably, MeaTech and Redefine Meat have developed 3D printers for meat products, with the former focusing more on cultured meat production.

Beyond print resolution and speed, sanitary equipment design is emerging as a critical selection criterion for commercial food printers, especially for meat, dairy, and ready-to-eat products. Key factors include the following: (1) ease of disassembly and cleaning of nozzles, tubes, and reservoirs; (2) use of food-grade materials (stainless-steel, FDA-approved polymers); (3) built-in temperature control to prevent bacterial growth in the ink holding system; and (4) UV or ozone sterilization options between print jobs. As food safety regulations tighten, we anticipate that future printer specifications will prominently feature hygienic ratings, similar to the IP ratings in industrial equipment. Therefore, when comparing commercial models, buyers should not only evaluate resolution and speed but also request validation data on cleanability and microbial reduction.

## 4. Conclusions, Challenge and Perspective

As an emerging additive manufacturing technology, 3D printing is increasingly favored by some manufacturing companies. This study reviews four types of 3D printing technologies in the food field: extrusion-based printing, inkjet printing, binder jetting and selective laser sintering. Among these, extrusion-based printing is dominant over the other types. Thus, 3D printing technology for personalized food preparation has become increasingly mature, and commercial 3D printers have been applied in many food sectors, including the preparation of chocolates, biscuits, candies, and coffee. In particular, 3D printers for personalized coffee and candy have been adopted in many industries, whereas printers for meat and meat products are still in the testing stage.

Based on the analysis of commercial printers and their respective ink property requirements, we propose the following general principles for printer selection, which are intended as practical guidelines rather than rigid rules. For materials with high viscosity exceeding 1000 Pa·s that exhibit good shear-thinning behavior, extrusion-based systems, whether piston-driven or screw-driven, remain the only technically feasible option. In contrast, for low-viscosity liquids below 100 mPa·s that require high-resolution surface decoration, inkjet printing is the preferred choice. When dealing with dry powder materials that can be agglomerated by a liquid binder, binder jetting offers a suitable solution, whereas heat-fusible powders such as sugars and starches that demand high production throughput are better suited for selective laser sintering. Special consideration should be given to temperature-sensitive materials, including proteins and living cells, for which cold extrusion with temperature-controlled printheads is strongly recommended. Furthermore, for applications involving multi-material or full-color printing, systems equipped with multiple extruders or hybrid platforms that combine inkjet and extrusion functionalities are advantageous. It must be emphasized that these principles are not absolute; the final selection of printing technology should also take into account practical factors such as equipment cost, production throughput, post-processing requirements, and hygiene constraints specific to food manufacturing environments.

Despite the remaining challenges, such as nutrient–ink interactions affecting printability, environmental impacts on product quality, and post-processing/packaging effects, future research in 3D food printing should concentrate on seven priority areas to realize its full potential. (1) Rheological ink design: establish standardized protocols (G′, G″, yield stress, recovery) and predictive models that link ink composition to printing performance, ensuring texture and stackability. (2) Hybrid printing systems: integrate multiple printheads (extrusion and inkjet) in one machine for simultaneous structural fabrication, surface decoration, and aroma/nutrient deposition. (3) Real-time quality monitoring: employ computer vision and AI-based feedback to adjust parameters during printing, minimizing defects and ensuring consistency. (4) Scalability and throughput: enhance printing speed and build volume, moving from prototyping to industrial production. (5) Sustainability: valorize food by-products (e.g., peels, grounds) as ink components, and develop biodegradable or edible packaging integrated with printed foods. (6) Regulatory and safety standards: define hygiene protocols for printhead cleaning, material handling, and pathogen control, especially for meat and dairy products. (7) Consumer acceptance: investigate how printed shapes and textures influence sensory perception and purchase willingness, facilitating co-creation and social engagement.

Addressing these directions will not only overcome current obstacles but also unlock the unique strengths of 3D food printing: personalized nutrition, diversified production, participatory food preparation, and new business and job creation. With continued interdisciplinary efforts, this technology will become a transformative pillar of future food systems.

## Figures and Tables

**Table 1:** Commercial 3D food printers for chocolate production. Complete information including pictures and websites is provided in Table S1 in the Supplementary Materials.

Manufacturer	3D Printer Model	Specification	Software/Supported File Formats	Extruder	Technical Type
Print2Taste GmbH, Koblenz, Germany	Procusini 5.0	Nozzle size: 0.5/1.3 mm	Procusini^®^ Club, G code	Single	Extrusion
Choc Edge Ltd., Exeter, UK	Choc Creator V2.0 Plus	Nozzle size: 0.4/0.8 mm, syringe volume: 30 mL, y-direction moving print bed	ChocPrint Slicing Program, G code	Single	FDM
FoodBot, Hangzhou, China	FoodBot S2 Multi ingredient Food 3D printer	Nozzle size: 0.4/1.55 mm	STL, G code	Single	Extrusion
Wiiboox, Shenzhen, China	WiibooxSweetin 3D chocolate printer	Nozzle size: 0.6/0.84 mm	STL, G code, OBJ, AMF	Single	FDM
Zmorph, Wrocław, Poland	ZMorph VX	Nozzle size: 0.2/0.4 mm	Voxelizer 2.0 STL, G code	Single/Dual extruder	Extrusion
3DCloud, Beijing, China	QiaoKe chocolate printer	Nozzle size: 0.8 mm	STL, G code	Single	Extrusion
Print4Taste GmbH, Koblenz, Germany	Mycusini 2.0	/	STL, G code	Single	Extrusion
Cocoa Press, Philadelphia, PA, USA	Cocoa Jet	Nozzle Size: 0.80 mm	STL, G code	Food Grade Steel	Extrusion
By Flow Focus, Eindhoven, The Netherlands	Focus 3D Food Printer	Nozzle Size: 0.30–1.60 mm	STL, G code	Single	Extrusion
3Desserts Graphiques, Lyon, France	The impressive	/	STL, G code	Dual extruder	Extrusion
Felix Food 3D Printer, IJsselstein, The Netherlands	FELIX TWIN Food 3D Printer	Nozzle Diameters (mm) 1.6, 2.5, 3.5	STL, G code	Dual extruder	Extrusion

STL: Stereolithography; OBJ: Object File Format; AMF: additive manufacturing file.

**Table 2:** Commercial 3D food printers for cakes, pizza, and cookies production. Complete information including pictures and websites is provided in Table S2 in the Supplementary Materials.

Manufacturer	3D Printer Model	Specification	Software/Supported File Formats	Technical Type
Natural machine, Barcelona, Spain	Foodini	Nozzle diameter(s) 0.5 mm	STL file	Extrusion
XYZPrinting, New Taipei City, China	XYZPrinting Food printer	Nozzle diameter(s) 1/2/4/8 mm	XYZware	Extrusion
PancakeBot, Hokksund, Norway	PancakeBot 2.0	/	Pancake Painter Software v1.4.0, FAT32	Extrusion
Creative Machine Lab, New York, NY, USA	Fab@Home Model 3.1	/	STL and XDFL	Extrusion
Zmorph, Wrocław, Poland	Zmorph 2.0 VX	/	.stl, .obj, .step, .dxf, .png, .bmp files	Dual Plastic Extruder and Thick Paste Extruder
Ultimaker, Utrecht, The Netherlands	Ultimaker S5	/	Ultimaker Essentials	Double extrusion
CreateBot, Zhengzhou, China	Createbot 3D Food Printer	Nozzle diameter(s) 0.4 mm–1.55 mm	STL file	Extrusion
Micromake, Zhengzhou, China	Food 3D printer	/	STL file	Extrusion
BeeHex, Austin, TX, USA	Chef 3D printer	/	STL file	Extrusion

STL: Stereolithography; XDFL: Extensible Digital Fabrication Language.

**Table 3:** Commercial 3D food printers for coffee. Complete information including pictures and websites is provided in Table S3 in the Supplementary Materials.

Manufacturer	3D Printer Model	Printing Speed/s	Resolution	Technical Type
Wiiboox, Shenzhen, China	Wiiboox Sweetin	10/20	640 × 480	Inkjet
MYJ, Shenzhen, China	COF + CISS	10/20	720 × 720 2880 × 1440 5760 × 1440	Inkjet
Colorato, Gronau, Germany	Coloranino^®^ Coffee Printer	5/10	/	Inkjet
ColorSun, Shenzhen, China	CSC5	15	600 × 600	Inkjet
Rainbow, Shenzhen, China	RB-04HP Selfie	10 (4 cups)	600 × 600	Inkjet

**Table 4:** Commercial 3D food printers for candy. Complete information including pictures and websites is provided in Table S4 in the Supplementary Materials.

Manufacturer	3D Printer Model	Specification	Software/Supported File Formats	Extruder	Technical Type
FoodBot, Hangzhou, China	FoodBot D2	Nozzle size: 0.4/1.5 mm	STL, G code, OBJ	Dual extruder	Extrusion
3D Systems, Rock Hill, SC, USA	ChefJet Pro	Layer thickness: 1 mm	STL	Single	Extrusion
Evil Mad Scientists Lab, Sunnyvale, CA, USA	CandyFab 6000	/	CandyFabulous	/	SHASAM
Katjes Fassin, Birmingham, UK	Magic Candy Factory	/	STL	Single	FDM

STL: Stereolithography; OBJ: Object File Format.

## Data Availability

No new data were created or analyzed in this study. Data sharing is not applicable to this article.

## Supplementary Materials

The following supporting information can be downloaded at: https://www.mdpi.com/article/10.3390/gels12090777/s1, Table S1: Complete information of commercial 3D food printers for chocolate production; Table S2: Complete information of commercial 3D food printers for cake, pizza, and cookie production; Table S3: Complete information of commercial 3D food printers for coffee; Table S4: Complete information of commercial 3D food printers for candy.
