# Supplementary material for: A Comparative Study of Commercial 3D Printers for Food Fabrication Based on Printing Ink Gels
Source: Gels. 2026 Aug 31;12(9):777. doi: 10.3390/gels12090777 (PMC13606077; doi:10.3390/gels12090777)
Supplement: Supplementary file 1 [file gels-12-00777-s001.zip › gels-4504424-supplementary.pdf]

Table S1. Complete information of commercial 3D food printers for chocolate production.

Manufacturer	3D printer model	Specification	Software/supported file formats	Extruder	Technical type	Picture of chocolate	Picture of printers	Reference/the linked website
Print2Taste GmbH, Koblenz, Germany	Procusini 5.0	Nozzle size: 0.5/1.3 mm	Procusini® Club, G code	Single	Extrusion	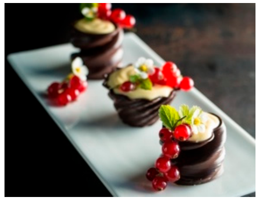	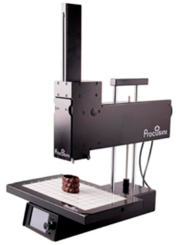	https://procusini.com/en/pages/procusini-5-0
Choc Edge Ltd., Exeter, UK	Choc Creator V2.0 Plus	Nozzle size: 0.4/0.8 mm, syringe volume: 30 ml, y-direction moving print bed	ChocPrint Slicing Program, G code	Single	FDM	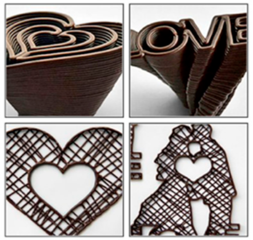	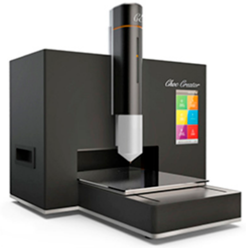	http://chocedge.com
FoodBot, Hangzhou, China	FoodBot S2 Multi ingredient Food 3D printer	Nozzle size: 0.4/1.55 mm	STL, G code	Single	Extrusion	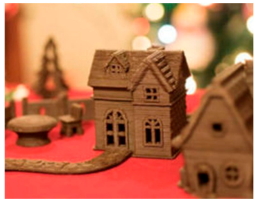	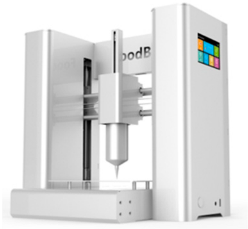	https://www.3dprintersonlinestore.com/

Manufacturer	3D printer model	Specification	Software/supported file formats	Extruder	Technical type	Picture of chocolate	Picture of printers	Reference/the linked website
Wiiboox, Shenzhen, China	WiibooxSweetin 3D chocolate printer	Nozzle size: 0.6/0.84 mm	STL, G code, OBJ, AMF	Single	FDM	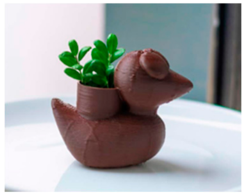	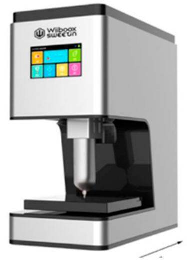	https://www.amazon.com/
Zmorph, Wrocław, Poland	ZMorph VX	Nozzle size:0.2/0.4 mm	Voxelizer 2.0 STL, G code	Single/Dual extruder	Extrusion	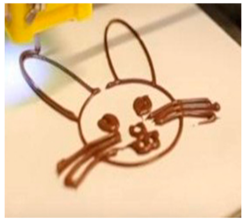	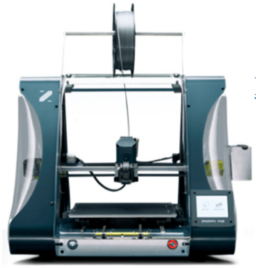	https://zmorph3d.com/
3DCloud, Beijing, China	QiaoKe chocolate printer	Nozzle size:0.8 mm	STL, G code	Single	Extrusion	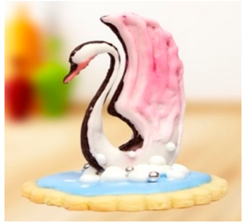	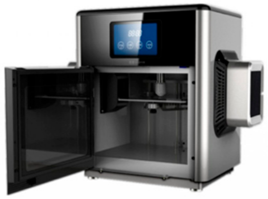	http://qiaoke.3dcloud.cn/

Manufacturer	3D printer model	Specification	Software/supported file formats	Extruder	Technical type	Picture of chocolate	Picture of printers	Reference/the linked website
Print4Taste GmbH, Koblenz, Germany	Mycusini 2.0	/	STL, G code	Single	Extrusion	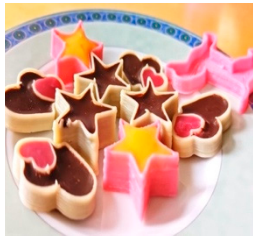	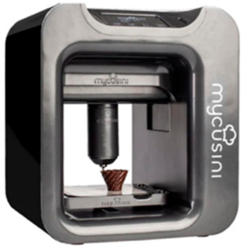	https://mycusini.com/en
Cocoa Press, Philadelphia, PA, USA	Cocoa Jet	Nozzle Size: 0.80mm	STL, G code	Food Grade Steel	Extrusion	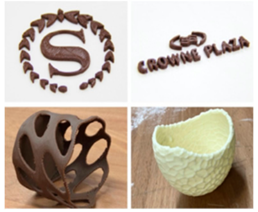	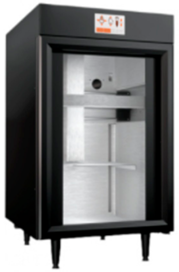	https://www.aniwaa.com/product/3d-printers/cocoa-press-cocoa-jet/
By Flow Focus, Eindhoven, The Netherlands	Focus 3D Food Printer	Nozzle Size: 0.30-1.60mm	STL, G code	Single	Extrusion	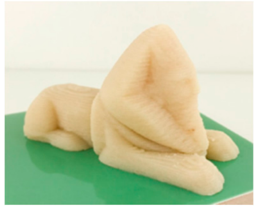	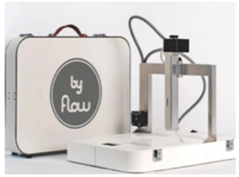	https://www.3dbyflow.com

Manufacturer	3D printer model	Specification	Software/supported file formats	Extruder	Technical type	Picture of chocolate	Picture of printers	Reference/the linked website
3Desserts Graphiques, Lyon, France	The impressive	/	STL, G code	Dual extruder	Extrusion	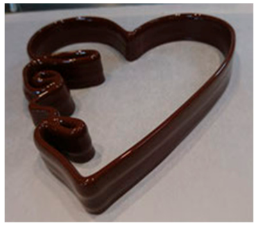	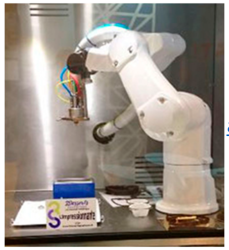	https://www.aniwaa.fr/produit/imprimantes-3d/3desserts-graphiques-limpressionnante/
Felix Food 3D Printer, IJsselstein, The Netherlands	FELIX TWIN Food 3D Printer	Nozzle Diameters (mm) 1.6, 2.5, 3.5	STL, G code	Dual extruder	Extrusion	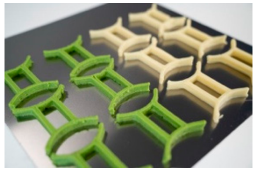	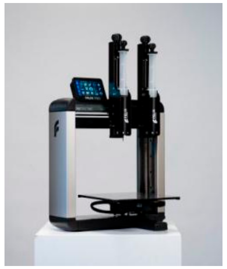	https://felixfood.nl/

Table S2. Complete information of commercial 3D food printers for cakes, pizza, and cookies production.

Manufacturer	3D printer model	Specification	Software/supported file formats	Technical type	Picture of printers	Reference/the linked website
Natural machine, Barcelona, Spain	Foodini	Nozzle diameter(s) 0.5mm	STL file	Extrusion	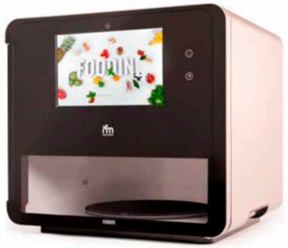	https://www.naturalmachines.com
XYZPrinting, New Taipei City, China	XYZPrinting Food printer	Nozzle diameter(s) 1/2/4/8 mm	XYZware	Extrusion	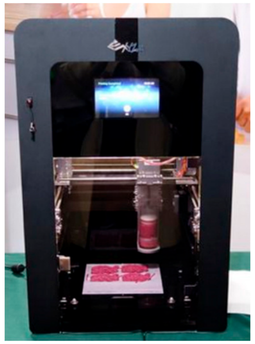	https://www.xyzprinting.com
PancakeBot, Hokksund, Norway	PancakeBot 2.0	/	Pancake Painter Software, FAT32	Extrusion	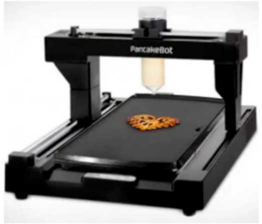	https://www.aniwaa.com/

Manufacturer	3D printer model	Specification	Software/supported file formats	Technical type	Picture of printers	Reference/the linked website
Creative Machine Lab,New York, NY, USA	Fab@Home Model 3.1	/	STL and XDFL	Extrusion	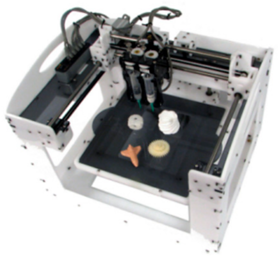	https://www.creativemachineslab.com/
Zmorph, Wrocław, Poland	Zmorph 2.0 VX	/	.stl, obj, step, .dxf, .png, .bmp files	Dual Plastic Extruder and Thick Paste Extruder	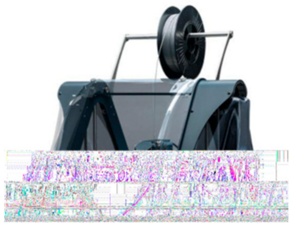	Thick Paste Extruder, Zmorph. https://zmorph3D.com/products/toolheads/thick-paste-extruder
Ultimaker, Utrecht, The Netherlands	Ultimaker S5	/	Ultimaker Essentials	Double extrusion	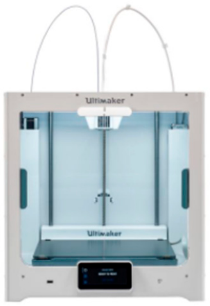	https://3deksperten.dk/ultimaker-5s.html

Manufacturer	3D printer model	Specification	Software/supported file formats	Technical type	Picture of printers	Reference/the linked website
CreateBot, Zhengzhou, China	Createbot 3D Food Printer	Nozzle diameter(s) 0.4 mm-1.55 mm	STL file	Extrusion	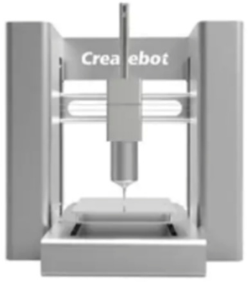	https://www.aniwaa.com/product/3d-printers/createbot-3d-food-printer/
Micromake, Zhengzhou, China	Food 3D printer	/	STL file	Extrusion	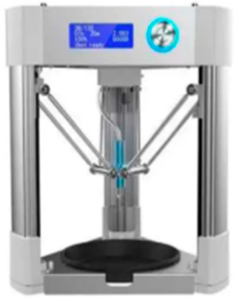	https://www.aniwaa.com/product/3d-printers/micromake-food-3d-printer/
BeeHex, Austin, TX, USA	Chef 3D printer	/	STL file	Extrusion	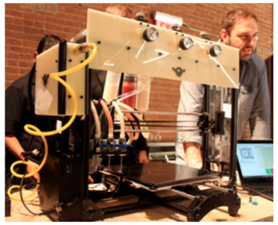	https://all3dp.com/2/3d-printed-pizza-myth-or-reality/

Table S3. Complete information of commercial 3D food printers for coffee.

Manufacturer	3D printer model	Printing speed/s	Resolution	Technical type	Picture of coffee	Picture of printers	Reference/the linked website
Wiiboox, Shenzhen, China	Wiiboox Sweetin	10/20	640*480	Inkjet	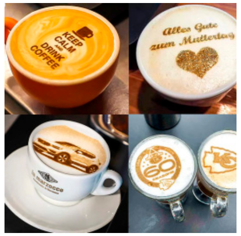	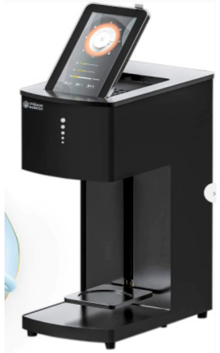	Wiiboox Sweetin Coffee Beverage 3D Printer – 3D Printers 2 U
MYJ, Shenzhen, China	COF+CISS	10/20	720*720<br>2880*1440<br>5760*1440	Inkjet	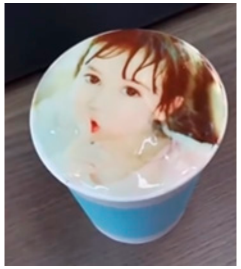	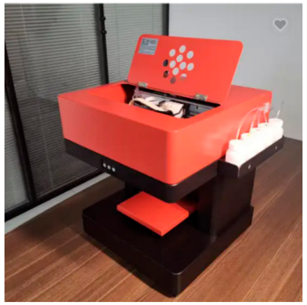	https://www.alibaba.com/product-detail/Automatic-Colorful-3D-Food-Cake-Coffee_1600173580443.html?spm=a2700.galleryofferlist.normal_offer.d_image.5b623456SeUKK2

Manufacturer	3D printer model	Printing speed/s	Resolution	Technical type	Picture of coffee	Picture of printers	Reference/the linked website
Colorato, Gronau, Germany	Coloranino® Coffee Printer	5/10	/	Inkjet	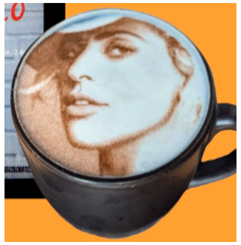	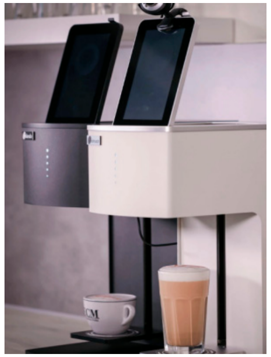	Coloranino® coffee printer - latte art printer - selfie coffee (coffeecolorato.com)
ColorSun, Shenzhen, China	CSC5	15	600*600	Inkjet	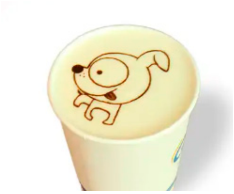	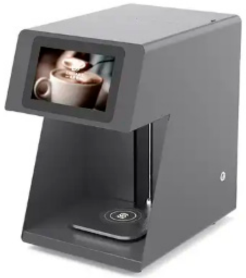	https://www.alibaba.com/product-detail/new-arrival-coffee-printer-machine-with_62376482790.html?spm=a2700.galleryofferlist.normal_offer.d_image.5ea93456mCV7G1

Manufacturer	3D printer model	Printing speed/s	Resolution	Technical type	Picture of coffee	Picture of printers	Reference/the linked website
Rainbow, Shenzhen, China	RB-04HP Selfie	10 (4 cups)	600*600	Inkjet	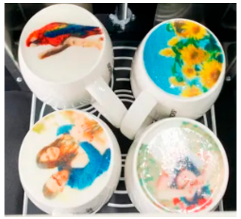	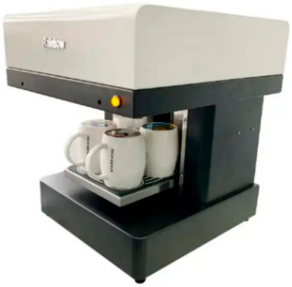	Edible Color 3d Coffee Art Printer For Food Macaron Printing Machine For Business - Buy 3d Coffee Art Printer,Coffee Art Printer,Coffe Printer Product on Alibaba.com

Table S4. Complete information of commercial 3D food printers for candy.

Manufacturer	3D printer model	Specification	Software/supported file formats	Extruder	Technical type	Picture of printers	Reference/the linked website
FoodBot, Hangzhou, China	FoodBot D2	Nozzle size: 0.4/1.5 mm	STL, G code,OBJ	Dual extruder	Extrusion	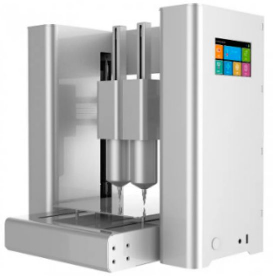	https://www.3dprintersonlinestore.com/foodbot-d2-food-3d-printer
3D Systems, Rock Hill, SC, USA	ChefJet Pro	Layer thickness: 1mm	STL	Single	Extrusion	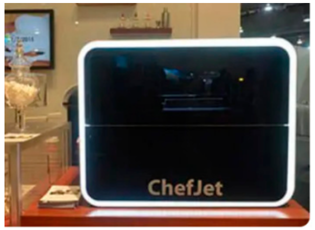	https://www.aniwaa.com/product/3d-printers/3d-systems-chefjet-pro/

Manufacturer	3D printer model	Specification	Software/supported file formats	Extruder	Technical type	Picture of printers	Reference/the linked website
Evil Mad Scientists Lab, Sunnyvale, CA, USA	CandyFab 6000	/	CandyFabulous	/	SHASAM	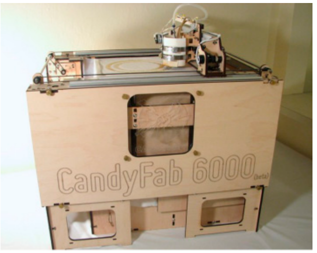	CandyFab丨A DIY 3D Sugar Printer
Katjes Fassin, Birmingham, UK	Magic Candy Factory	/	STL	Single	FDM	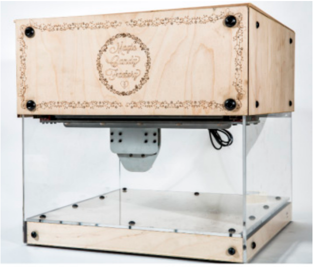	https://3dprint.com/122184/magic-candy-factory/
